# Analysis of Niobium and Stainless Steel Electropolishing Solutions by Laser-Induced Breakdown Spectroscopy Using a Porous Silicon Substrate and a Non-Contact Pretreatment

**DOI:** 10.3390/ma19030637

**Published:** 2026-02-06

**Authors:** Ayumu Matsumoto, Yuki Takeda, Kiichi Kuroda, Hiroto Torigoe, Yui Sugita, Yusuke Shimazu, Keisuke Nii, Yoshiaki Ida, Shinji Yae

**Affiliations:** 1 Department of Chemical Engineering and Materials Science, Graduate School of Engineering, University of Hyogo, 2167 Shosha, Himeji 671-2280, Japanyae@eng.u-hyogo.ac.jp (S.Y.); 2 Marui Galvanizing Co., Ltd., 402 Kou, Shirahama-cho, Himeji 672-8023, Japan; keisuke_nii@e-marui.jp (K.N.); yoshiaki_ida@e-marui.jp (Y.I.)

**Keywords:** surface-enhanced LIBS, metal-assisted chemical etching, silicon nanostructures, microvolume analysis, sample pretreatment, industrial process monitoring

## Abstract

Electropolishing is an essential process for the surface treatment of metallic materials. To determine the appropriate replacement timing of electropolishing solutions for their efficient use and improved productivity, it is important to periodically analyze the amounts of dissolved metals in the solutions. However, these solutions are typically highly corrosive, and on-site analytical techniques that can be easily applied at production sites have not yet been established. In this study, we demonstrated microvolume liquid analysis using low-energy laser-induced breakdown spectroscopy (LIBS) combined with a porous silicon substrate fabricated by metal-assisted etching (metal-assisted chemical etching) and a non-contact gas-blowing pretreatment. In the analysis of electropolishing solutions used for niobium superconducting cavities and stainless steel products, emission lines of niobium and of iron and chromium were successfully detected after blowing the respective microdroplet samples on porous silicon, and linear correlations were observed between the spectral line intensity and the polished amounts. The present results provide a basis for future on-site application of LIBS to highly corrosive electropolishing solutions in the metal finishing industry.

## 1. Introduction

Electropolishing is an essential surface finishing process for metallic materials used in various fields [1,2]. In this process, a metal workpiece is anodically polarized to sufficiently positive potentials in concentrated acids, leading to electrochemical dissolution of the material surface without introducing mechanical or thermal damage. Under optimized conditions, an extremely smooth surface can be obtained.

Niobium (Nb) is planned to be used as the material for superconducting radio-frequency (SRF) cavities in the International Linear Collider (ILC) [3,4,5], a next-generation large-scale particle accelerator, because of its superior superconducting properties, such as a relatively high critical temperature and low surface resistance, together with high thermal conductivity. Electropolishing is essential to obtain a highly smooth inner surface of Nb cavities for efficient particle acceleration [6,7]. The electropolishing solution for Nb is typically composed of hydrofluoric acid (HF) and sulfuric acid (H_2_SO_4_) [8], and anodic dissolution is interpreted to proceed via oxide formation followed by chemical dissolution of the oxides by fluoride species [9]. Since mass production of high-quality Nb cavities will be required for the ILC [5], a cost-effective surface treatment process needs to be developed. To address this issue, various studies have investigated anodic dissolution behavior, surface products, and dissolved species [6,9,10,11,12], and in parallel, efforts have been made to improve electropolishing systems [13,14,15,16].

Stainless steels are the most extensively electropolished materials in industry [1,17]. Because of their high corrosion resistance, austenitic stainless steels such as SUS304 and SUS316L are widely used in pharmaceutical, food, and semiconductor facilities. The improved corrosion resistance is achieved by electropolishing, as it removes the mechanically damaged layer and produces a chromium (Cr)-enriched passive surface [18,19,20]. Electropolishing solutions for these materials are typically based on phosphoric acid (H_3_PO_4_) and H_2_SO_4_, and the dissolution behavior has been investigated from various aspects [21,22,23,24,25], including the formation of a viscous layer [2].

In these applications, electropolishing solutions must be periodically replaced, as the polishing accuracy deteriorates with repeated use. The polished amount can be an indicator to determine the appropriate replacement timing for efficient use and improved yield, and its monitoring can also be useful for anomaly detection. However, it is difficult to handle the highly corrosive electropolishing solutions. In wet chemical processes, metal concentrations are usually measured by laboratory-based instruments such as inductively coupled plasma atomic emission spectroscopy (ICP-AES) and atomic absorption spectroscopy (AAS). Although highly sensitive and accurate quantification can be achieved, these methods typically require sampling of a certain volume of solution, transportation to a laboratory, and sufficient dilution to mitigate matrix effects and to prevent damage to instrument components, thereby hindering on-site applications. Online measurement systems are being developed, but their applications are still limited, and the installation costs remain high. For these reasons, it is desirable to develop simple systems that can be easily applied to on-site analysis of highly corrosive solutions at production sites.

Laser-induced breakdown spectroscopy (LIBS) is an analytical technique that measures the emission spectra of a plasma generated by laser ablation [26,27,28]. Owing to its capability for rapid elemental identification with a simple optical setup, LIBS has attracted attention as a promising technique in extreme environments, such as space [29,30], the deep sea [31,32,33], and nuclear decommissioning sites [34,35,36,37], as well as for industrial process monitoring [38,39,40,41,42,43]. While LIBS instruments for analyzing solid samples have been commercially developed, the establishment of reliable liquid analysis systems remains an important issue.

Many approaches for effectively analyzing liquid samples have been reported [44,45], beginning with spectroscopic analysis of laser-induced plasmas generated inside liquids [46]. Among various approaches, such as filter paper absorption [47,48], liquid jet analysis [49,50,51], solid substrate immersion [52,53,54], and electrodeposition [55,56], we have focused on surface-enhanced LIBS [57]. In this method, a sample droplet placed on a solid substrate is dried, and the resulting dry residue is analyzed by LIBS. In addition to flat substrates [57,58,59], diverse substrates have been examined, such as patterned [60,61,62], nanoparticle-dispersed [63], wettability-controlled [64,65], and porous [66,67,68] substrates. While various types of liquids, including wine [69], oil [70], and blood [71], can be analyzed, this method is especially suitable for hazardous samples as it requires only a microvolume of sample. Compared with conventional laboratory-based techniques such as ICP-AES and AAS, surface-enhanced LIBS enables rapid analysis without complex pretreatment.

We have reported the use of a silicon (Si) substrate with a porous layer (porous Si substrate), aiming at the analysis of dissolved elements in toxic aqueous solutions [72,73,74,75], where porous Si was fabricated by displacement deposition of metal nanoparticles [76] followed by metal-assisted etching (metal-assisted chemical etching) [77,78,79,80,81,82]. Various factors should be considered when selecting substrates for surface-enhanced LIBS, such as material and fabrication costs, spectral interference, plasma generation efficiency, and sample retention. Among the various substrates, porous Si was chosen in this study because it offers the following advantages. Since porous structures can be uniformly formed over a large area through simple solution-immersion processes, the substrates can be obtained at low cost. In the visible wavelength range, the number of intense Si atomic (ionic) emission lines is limited, and band emissions from the molecules formed in a Si plasma in air are relatively weak. These features allow the detection of target elements with minimal spectral interference from the substrate. The porous structure enhances the signal intensity and stability through increased laser absorption [83] due to multiple reflections between the pore walls (Si absorbs 1064 nm light commonly used in LIBS) and through suppression of inhomogeneous deposition of dry residue [73] due to infiltration of the sample solution into the pores. The enhanced laser absorption can also facilitate plasma generation at lower laser energies, which is advantageous for developing compact and portable LIBS systems for on-site applications.

In our previous study [75], we analyzed Nb electropolishing solutions by LIBS using porous Si. A key issue is the high concentration of the electropolishing solution, as it is replaced when the Nb concentration reaches approximately 10 g/L at the test facility [11]. Under such conditions, a thick dry residue is formed, which disturbs the plasma generation. Therefore, we preliminarily wiped the excess solution on the substrate with paper, which enabled the estimation of the polished amounts in the electropolishing solutions while suppressing excessive deposition of dry residue. However, contamination originating from wiping paper, such as calcium (Ca), affected the observed spectra, which is also undesirable for assessing impurities originally present in the sample solutions.

In this study, we analyzed highly corrosive electropolishing solutions using a non-contact gas-blowing pretreatment on porous Si. The key novelty is that gas blowing was combined with capillary infiltration into the porous layer, which suppressed the formation of a thick dry residue from highly concentrated Nb electropolishing solutions while retaining an appropriate amount of sample for LIBS detection, without introducing contaminants from contact materials. We also extended this approach from Nb electropolishing solutions to stainless steel electropolishing solutions, providing an early demonstration of LIBS applied to electropolishing for stainless steel products, which are widely used in industry.

## 2. Materials and Methods

### 2.1. Substrate Fabrication

**Figure 1:**
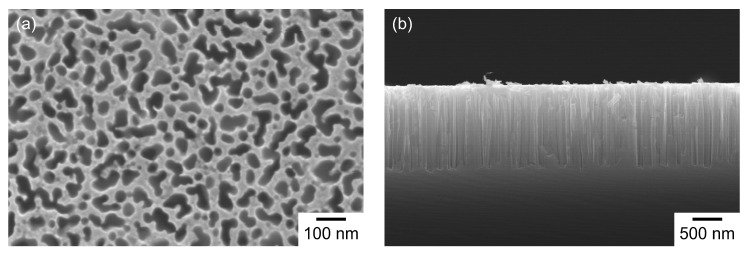
(**a**) Surface and (**b**) cross-sectional SEM images of an etched region of a substrate used in this study.

Metal-assisted etching was conducted in a circular region with a diameter of approximately 6 mm at the center of a 15 mm × 15 mm square Si substrate. Figure 1 shows surface and cross-sectional images of an etched region of a substrate used in this study, which were taken with a scanning electron microscope (SEM) (JEOL Ltd., Tokyo, Japan, JSM-7001F). On the substrate surface (Figure 1a), pores with sizes on the order of several tens to approximately 100 nm were observed, and the porosity estimated from a binarized image was approximately 45%. From the cross-sectional view (Figure 1b), a porous layer with a thickness of approximately 1.4 μm was observed, in which vertically aligned pores were formed. This porous structure enabled successful Nb detection in the electropolishing solution samples in our previous study using the wiping pretreatment [75]. Therefore, the same substrate fabrication conditions were used in this study to allow a direct comparison between the gas-blowing and wiping methods. From a physical perspective, such small pores can facilitate liquid infiltration into the porous layer, since the capillary pressure increases as the pore radius decreases. In addition, a porous layer thickness on the micrometer scale can provide sufficient pore volume to retain enough sample for LIBS detection. Assuming that the effective ablation depth is in the sub-micrometer to approximately 1 μm range [54,72], the porous layer thickness is expected to allow ablation of the porous layer containing the retained sample rather than ablating the underlying dense Si.

For porous Si formation, an n-type Si (100) wafer (resistivity: 0.5–10 Ω·cm, thickness: 725 ± 25 μm) was used as the starting substrate. After pretreatment [81] including the CP-4A method [76], the substrate was immersed in an aqueous solution containing 0.15 M (M: mol/L) hydrofluoric acid (HF) and 0.35 mM silver(I) nitrate at 298 K for 600 s to deposit silver (Ag) nanoparticles, which act as catalysts for subsequent etching. The Ag-deposited substrate was immersed in an etching solution at 298 K for 120 s, resulting in selective dissolution of the Si surface directly beneath the Ag nanoparticles. The etching solution was prepared by mixing an aqueous solution containing 6.6 M HF and 0.1 M hydrogen peroxide with ethanol (≥99.5%) at a volume ratio of 20:1. A masking tape with a circular hole with a diameter of approximately 6 mm was attached to the substrate during etching to limit the etched region. The etched substrate was immersed in 60% nitric acid for 600 s at room temperature to form a thin silicon dioxide layer (approximately 1 nm) [84], which offers a hydrophilic surface and can facilitate the infiltration of aqueous samples into the pores. The Ag catalysts were inevitably dissolved in this process. The substrate was rinsed with ultrapure water and dried with nitrogen (N_2_) gas blowing after each treatment.

### 2.2. Sample Preparation

In this study, electropolishing solution samples prepared at an industrial production site were used. These samples were synthetic Nb electropolishing solutions with a representative acid composition, obtained by dissolving Nb via electropolishing of a Nb plate in a HF-H_2_SO_4_ mixture (55% HF:98% H_2_SO_4_ = 1:9 in volume). The polished amount (g/L) was defined as the weight loss of the metal plate divided by the initial solution volume. Assuming that the solution is replaced when the Nb concentration reaches approximately 10 g/L in the test facility described in Ref. [11], sample solutions with polished amounts of 1.0, 2.5, 5.0, and 10.0 g/L were prepared. This range includes the assumed replacement criterion and lower values for early-stage monitoring and for assessing the linearity of the spectral line intensity with respect to the polished amount. Note that the polished amount represents a nominal concentration and is not strictly equivalent to the dissolved metal concentration in the solution. Since H_2_SO_4_ is not expected to be stoichiometrically consumed in the primary Nb dissolution pathway [9] and the solution was stored under sealed conditions, volume change was assumed to be negligible. For stainless steel electropolishing solution samples, an SUS316L plate (carbon: 0.008, Si: 0.67, manganese: 0.88, phosphorus: 0.035, sulfur: 0.002, nickel (Ni): 12.13, Cr: 17.41, molybdenum (Mo): 2.06, cobalt: 0.28, and iron (Fe): balance; all in wt.%) was electropolished in a H_3_PO_4_-H_2_SO_4_ electropolishing solution (85% H_3_PO_4_:98% H_2_SO_4_ = 3:1 in volume). For consistency with the Nb samples, the polished amounts were set to a similar range of 1.0, 3.1, 5.5, and 10.0 g/L.

**Figure 2:**
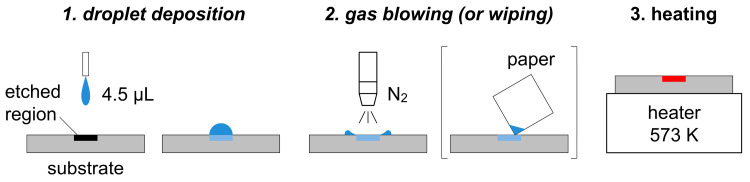
Sample preparation procedure through droplet deposition, gas blowing (or wiping), and heating.

Figure 2 shows the procedure for sample preparation. A 4.5 μL sample droplet was placed on the substrate using a micropipette (Eppendorf SE, Hamburg, Germany, 0.5–10 μL), which spread over the circular etched region. After standing for 120 s, N_2_ gas was blown onto the substrate using a compressed gas gun (AS ONE Corporation, Osaka, Japan, CAGN-P-14) connected to a N_2_ gas cylinder (output pressure: approximately 0.3 MPa). The distance between the gas nozzle and the substrate, the blowing angle relative to the substrate surface, and the blowing duration were approximately 3 cm, 90°, and 5 s, respectively. After this treatment, no visible liquid remained on the substrate. During gas blowing, the substrate was held with tweezers, and the surrounding area was protected from liquid scattering. While the pretreatment was carried out following the same procedure, minor operator-dependent variation may remain. For reliable on-site applications, the use of simple jigs or automation will be considered to further improve consistency. As a reference, the wiping process examined in the previous study [75] is also illustrated in Figure 2, and the resulting spectrum is used for comparison in the Section 3. After the excess solution on the substrate was removed, it was heated for 60 s on a heater set to 573 K to promote the removal of volatile components. The operations above were conducted in a fume hood at room temperature.

### 2.3. LIBS

**Figure 3:**
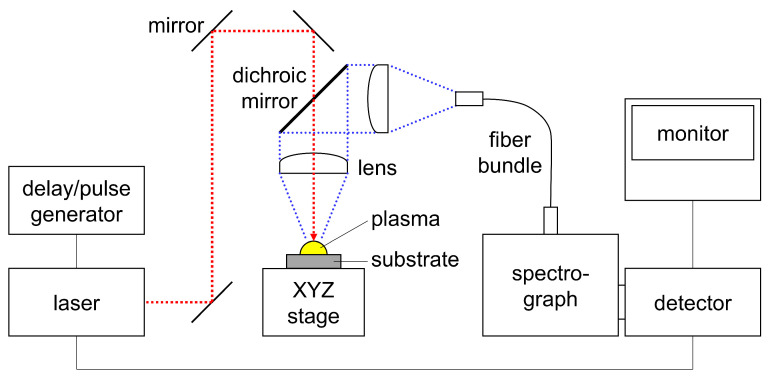
Experimental system for LIBS.

Figure 3 shows the experimental system for LIBS. The system is the same as that used in the previous study [75], and the schematic illustration has been updated. A Nd:YAG pulsed laser (Continuum, San Jose, CA, USA, Minilite II) with a wavelength of 1064 nm and a pulse duration of approximately 10 ns was focused onto a substrate placed on an XYZ stage through a long-pass dichroic mirror and an achromatic lens with a focal length of 75 mm, producing a plasma plume on the substrate. For the analysis of Nb electropolishing solutions, the pulse energy was 2.0 mJ. The plasma emission was introduced to a spectrograph (Bunkoukeiki Co., Ltd., Tokyo, Japan, M25-TPK1) through the achromatic lens, dichroic mirror, another achromatic lens, and an optical fiber bundle. The entrance slit width of the spectrograph was 50 µm, and a grating with a groove density of 1200 grooves/mm and a blaze wavelength of 500 nm was used. Emission spectra of the plasma were acquired using an intensified charge-coupled device (ICCD) detector (Princeton Instruments, Inc., Trenton, NJ, USA, PI-MAX: 1K) with an image intensifier gain of 100, a gate delay of 1.5 µs from the external trigger signal (Q-switch output signal of the laser source), a gate width of 5.0 µs, and a vertical binning height of 200 pixels. The detection timing was chosen, following the basic principles of LIBS [26,27], to avoid strong continuum emission and self-absorption of spectral lines in the early-stage plasma, as well as broadband molecular emission in the later-stage plasma, while ensuring sufficient emission line intensity. These conditions are the same as those used in the previous study [75]. For the analysis of stainless steel electropolishing solutions, the pulse energy was increased to 4.0 mJ, and a grating with a blaze wavelength of 200 nm was used to achieve sufficient signal intensity.

The laser repetition rate was controlled by a digital delay/pulse generator (Stanford Research Systems, DG645, Sunnyvale, CA, USA) and set to 0.3 Hz. The substrate stage was manually moved shot by shot with a step width of 0.2 mm to scan the laser irradiation position in one direction across the etched region with a diameter of approximately 6 mm. Dark spectra were acquired without laser irradiation, and the emission spectra after subtracting the dark spectra were used for analysis. For each substrate, one line scan was conducted, and an average spectrum on the etched region was obtained. The spectral data used to generate the figures in the present study are provided in the Supplementary Materials.

## 3. Results and Discussion

### 3.1. Analysis of Nb Electropolishing Solutions

**Figure 4:**
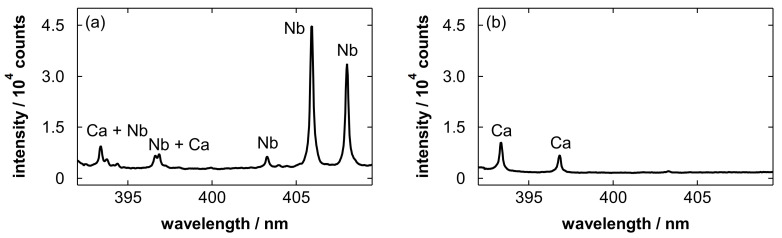
Emission spectra obtained after blowing Nb electropolishing solutions with polished amounts of (**a**) 5.0 and (**b**) 0 g/L. Major emission lines of Nb I at 393.74, 396.61, 403.25, 405.89, and 407.97 nm, and Ca II at 393.37 and 396.85 nm are labeled in the spectra.

Figure 4 shows emission spectra obtained after blowing Nb electropolishing solutions with polished amounts of 5.0 and 0 g/L (blank). The blank solution was the electropolishing solution without the Nb dissolution step. In Figure 4a, emission lines of Nb atoms [85] originating from the sample solution were clearly observed. This implies that an appropriate amount of sample for LIBS detection remained in the porous layer after gas blowing. In addition to Nb, the Ca ionic lines at 393.37 and 396.85 nm [86] overlapping with the Nb lines were observed. In the analysis of the blank sample (Figure 4b), only the Ca lines were observed.

**Figure 5:**
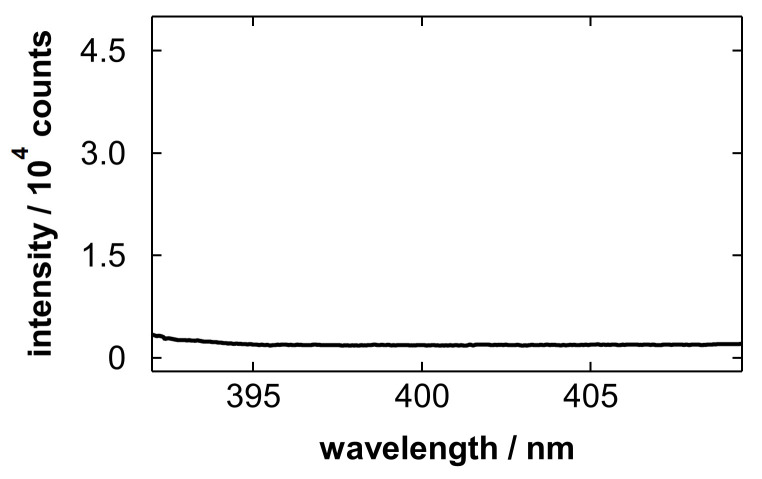
Emission spectrum obtained without sample deposition.

Figure 5 shows an emission spectrum obtained without sample deposition. Comparing Figure 4 with Figure 5, it can be found that spectral interference from the substrate is negligible in this wavelength range. The fact that the Ca lines were observed even for the blank solution while no distinguishable lines were observed without sample deposition, suggests that the Ca signal is attributable to impurities in the solution, rather than to the substrate or the electropolishing process. Possible sources of Ca contamination include trace impurities introduced during solution preparation at an industrial production site for electropolishing.

**Figure 6:**
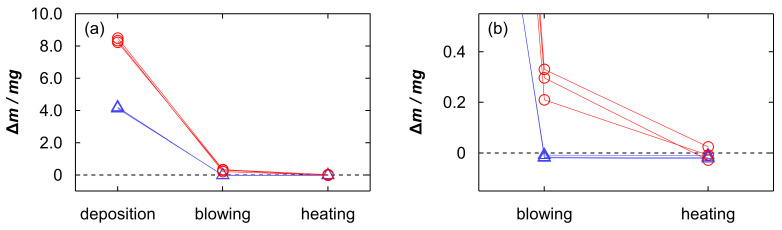
Weight changes (Δ*m*) of the substrate caused by depositing a 4.5 µL Nb electropolishing solution with a polished amount of 5.0 g/L or water, followed by gas blowing and heating. The substrate weight after droplet deposition was recorded at 30 s after placing the droplet, and all plots represent the weight change relative to the initial substrate weight before droplet deposition. Red circles and blue triangles indicate the Nb solution and water, respectively. Three replicate measurements are shown for each sample, and data points from the same replicate are connected by lines in the corresponding colors. The dashed line indicates Δ*m* = 0. (**a**) Overall and (**b**) enlarged views.

We additionally investigated the retention of the sample solution in the porous layer from the viewpoint of the substrate weight, which was measured using a microbalance (Mettler-Toledo International Inc., XP2U, Columbus, OH, USA). Figure 6 shows the weight changes caused by the droplet deposition of a 4.5 µL Nb electropolishing solution with a polished amount of 5.0 g/L and subsequent processes. During this experiment, the substrate weight increased gradually with time after placing the sample droplet. This weight increase can be attributed to the hygroscopic nature of concentrated H_2_SO_4_, which absorbs moisture from the atmosphere. Hence, we recorded the weight at a constant time of 30 s after placing the droplet. The weight increased by approximately 8.4 mg by depositing the sample, which is reasonable considering that the density of the electropolishing solution is approximately 1.8 g/cm^3^ and the hygroscopic nature. After gas blowing, the substrate weight was considerably reduced (Figure 6a). However, the weight change relative to the initial substrate remained positive (Figure 6b), although some variation was observed. This indicates that a portion of the sample solution remained on the substrate. The heating process further decreased the substrate weight, and the weight change became close to zero within the experimental uncertainty, suggesting that the remaining solution was largely removed by heating. Note that the amount of Nb contained in the 4.5 µL droplet (5.0 g/L) is only 0.0225 mg, and the residual Nb after gas blowing would be sufficiently small to fall within the experimental uncertainty of the weight measurement. As a reference, we added the weight measurement results for water droplet to the graph. In contrast to the electropolishing solution, the substrate weight decreased gradually with time after placing a 4.5 µL water droplet, possibly due to evaporation, and the weight increase measured after 30 s was approximately 4.3 mg. After gas blowing, the weight change relative to the initial substrate became zero, and it did not vary by heating. This suggests that the deposited water was almost completely removed by gas blowing. The remaining electropolishing solution after gas blowing can be attributed to the high viscosity and low volatility of the H_2_SO_4_-based solution. Although the detailed state and spatial distribution of the residual materials remain unclear, the weight measurements support that the sample was trapped in the porous layer even after gas blowing.

**Figure 7:**
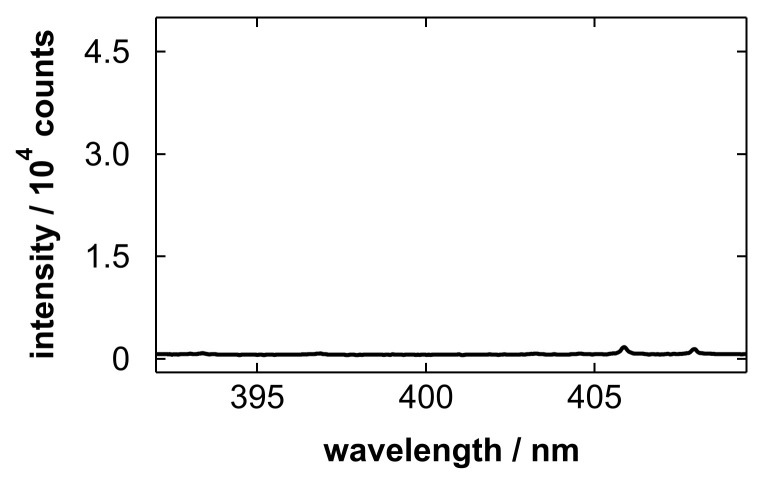
Emission spectrum obtained after blowing a Nb electropolishing solution with a polished amount of 5.0 g/L without subsequent heating.

We also conducted LIBS analysis of an unheated substrate. Figure 7 shows an emission spectrum acquired on the porous region after gas blowing without subsequent heating. In this case, emission lines were barely observed. This is likely because the wet surface and remaining liquid in the porous layer reduced the efficiency of plasma generation. This result supports that the heating process is important for LIBS detection of dissolved metals after gas blowing.

**Figure 8:**
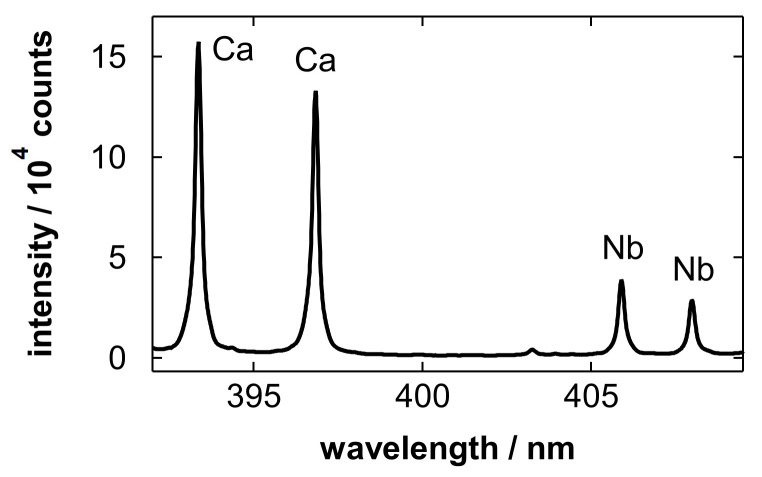
Emission spectrum obtained after wiping a Nb electropolishing solution with a polished amount of 5.0 g/L. Major emission lines of Nb I at 405.89 and 407.97 nm, and Ca II at 393.37 and 396.85 nm are labeled in the spectra. Reproduced from Ref. [75] with permission from The Royal Society of Chemistry.

Figure 8 shows an emission spectrum obtained after wiping the Nb electropolishing solution [75]. The intensity of the Ca lines obtained through wiping (Figure 8) was considerably higher than that obtained through gas blowing (Figure 4). The Ca content of the wiping paper can be dissolved into the electropolishing solution during the wiping process, and it remains on the substrate and affects the observed spectra. This difference suggests that the use of gas blowing enables the detection of impurities originally contained in the sample solution without disturbance from the elements of wiping paper, which is one of the advantages of non-contact sample pretreatment. Note that the wiping process might introduce not only Ca but also other inorganic elements, such as magnesium and potassium, from the ash content of the pulp-based wiping paper. As possible impurities in electropolishing solutions at production sites, in addition to Ca, sodium, magnesium, chlorine, and potassium can be introduced via residual contamination on tanks and piping, as well as from handling processes.

**Figure 9:**
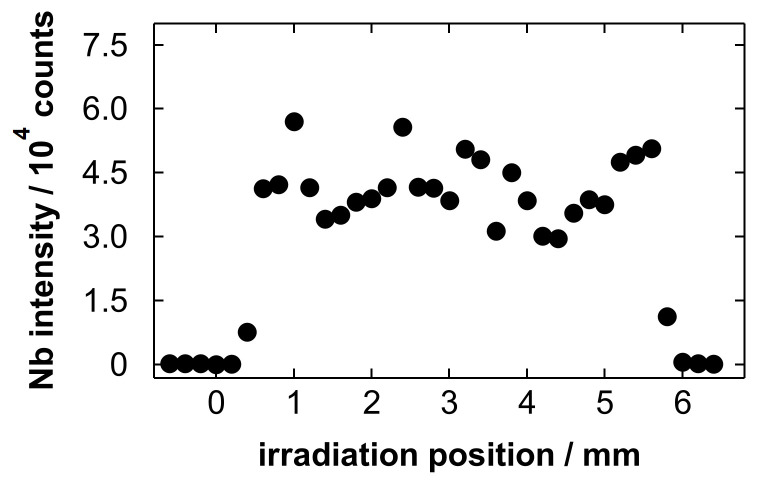
Line scan profile of the Nb intensity at 405.89 nm obtained after blowing a Nb electropolishing solution with a polished amount of 5.0 g/L.

Figure 9 shows a line scan profile of the intensity of the Nb emission at 405.89 nm obtained after gas blowing. In this study, we determined the spectral line intensity by subtracting the background intensity from the peak height. The background intensity was obtained as the average intensity at a certain wavelength range near the targeted line. The wavelength range for the background determination of the Nb line was 404.02 to 404.98 nm (26 points). We started the line scan analysis at an unetched region, and the Nb emission was detected when irradiating the etched region. The diameter of the irradiation craters was approximately 0.13 mm [75], and they did not overlap each other. The width of the Nb detected region was approximately 6 mm, which corresponds to the diameter of the etched region. This implies that the sample droplet spread over the etched region, and a portion of the sample was trapped in the porous layer. The relative standard deviation (RSD) value of the Nb intensity at the strongly detected region in Figure 9 (from 0.6 to 5.6 mm) was 17.5%. This suggests that the residual Nb was distributed on the substrate with a certain uniformity after gas blowing. The average intensity within this region was used as a representative value for constructing the calibration curve shown in the next paragraph.

**Figure 10:**
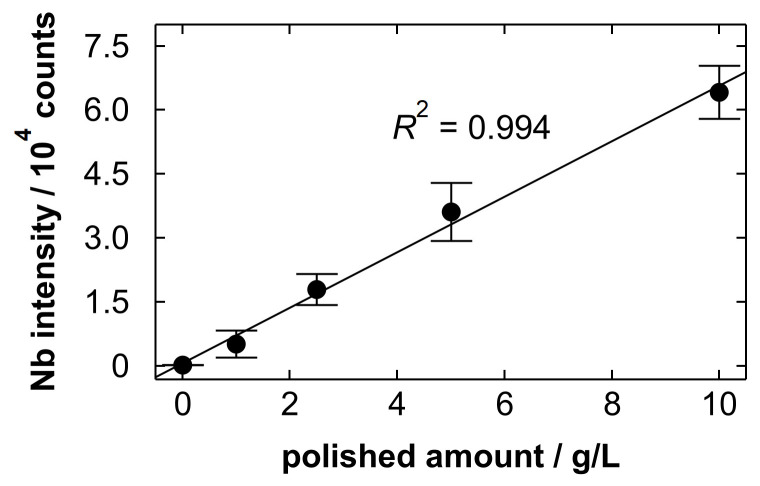
The Nb intensity at 405.89 nm plotted as a function of polished amount in the analysis of Nb electropolishing solutions through gas blowing. Each point represents the average intensity of three replicate measurements, and error bars indicate the standard deviation. A linear function fitted to the average intensity is shown together with its *R*^2^ value.

We analyzed Nb electropolishing solutions with different polished amounts of 0, 1.0, 2.5, 5.0 and 10.0 g/L through gas blowing. Figure 10 shows the Nb intensity plotted as a function of polished amount. The Nb intensity increased linearly with polished amount. This implies that the sample volume left after gas blowing did not change depending on the concentration of Nb in the droplet and that the residual Nb increased in proportion to the Nb concentration. The intensity did not exhibit saturation behavior, suggesting that self-absorption [87] is limited under the present conditions. The coefficient of determination (*R*^2^) value of the linear function fitted to the average intensity of three replicate measurements was 0.994. This suggests that the polished amount can be estimated by the classical calibration method. The limit of detection (LOD) and limit of quantification (LOQ) were 0.13 and 0.44 g/L, respectively, which were calculated from the following equations: LOD = 3*σ*/*S* and LOQ = 10*σ*/*S* where *σ* is the standard deviation of the background noise and *S* is the slope of the calibration curve. The background noise was evaluated over the wavelength range from 405.70 to 406.08 nm (11 points) using the blank spectrum with the maximum *σ* among three measurements. These values are sufficient to determine the replacement timing of the solution based on the polished amount of the workpiece (approximately 10 g/L). Note that the detection limit is significantly higher than that in the previous study [74] analyzing low-concentration solutions (LOD = 0.67 µg/L for strontium), because most of the deposited droplet was intentionally removed by gas blowing for the analysis of high-concentration components. At the polished amount of 10.0 g/L, the RSD value of the Nb intensity evaluated based on three replicate measurements was 9.8%. Here, we roughly estimate a minimal detectable change in polished amount around the assumed criterion. At 10.0 g/L, the standard error of the Nb intensity from three replicate measurements was calculated to be 3600 counts by dividing the standard deviation by the square root of three. The corresponding standard error in polished amount was then estimated to be 0.56 g/L by dividing this intensity error by the slope of the calibration curve. When comparing two independent values with the same standard error, the standard error of their difference is that of each value multiplied by the square root of two. Using a normal approximation (95% confidence), the detectable change corresponds to 1.96 times this value, i.e., approximately 1.5 g/L. While the detectable change is not small, it is considered sufficient for trend monitoring around the assumed replacement criterion. For other applications requiring higher accuracy, further improvements will be required, such as intensity normalization and multivariate analysis using additional spectral information.

### 3.2. Analysis of Stainless Steel Electropolishing Solutions

**Figure 11:**
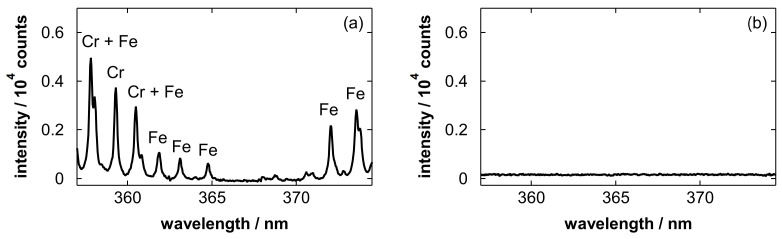
Emission spectra obtained after blowing stainless steel electropolishing solutions with polished amounts of (**a**) 5.5 and (**b**) 0 g/L. Major emission lines of Fe I at 358.12, 360.89, 361.88, 363.15, 364.78, 371.99, 373.49, and 373.71 nm (the last two lines overlap and are labeled together as Fe), and Cr I at 357.87, 359.35, and 360.53 nm are labeled in the spectra.

Figure 11 shows emission spectra obtained after blowing stainless steel electropolishing solutions with polished amounts of 5.5 and 0 g/L (blank). In Figure 11a, Fe and Cr emission lines [86] were simultaneously observed, which are attributed to the elements dissolved from SUS316L. This result demonstrates the applicability of the proposed method to the analysis of electropolishing solutions for stainless steels. As can be seen in Figure 11b, spectral interference from the substrate was negligible in this wavelength range. Note that the pulse energy was increased to 4.0 mJ because the Fe and Cr emission lines were not sufficiently intense at 2.0 mJ. This is likely due to the low sensitivity of the detection system in the UV range, as well as the high viscosity and low volatility of the H_3_PO_4_-based solution, which may reduce the effectiveness of liquid removal by gas blowing and heating.

**Figure 12:**
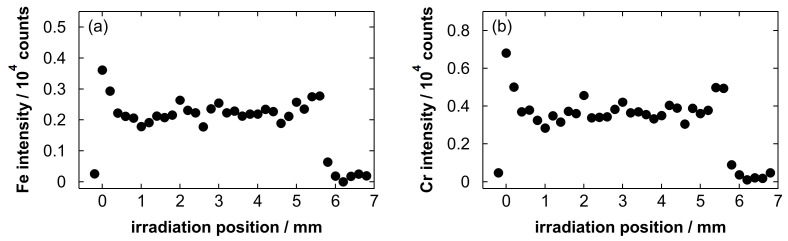
Line scan profiles of (**a**) the Fe intensity at 371.99 nm and (**b**) the Cr intensity at 359.35 nm obtained after blowing a stainless steel electropolishing solution with a polished amount of 5.5 g/L.

Figure 12 shows line scan profiles of the intensity of the Fe emission at 371.99 nm and the Cr emission at 359.35 nm. The wavelength ranges for the background determination of the Fe and Cr lines were 369.52 to 369.97 nm (13 points) and 366.00 to 366.79 nm (22 points), respectively. The intensity of both emission lines exhibited similar profiles, and the RSD values of the Fe intensity and the Cr intensity at the strongly detected region in Figure 12 (from 0 to 5.6 mm) were 16.4% and 20.3%, respectively. This suggests that the residual Fe and Cr were distributed on the substrate with a certain uniformity, showing similar spatial distributions. The average intensity within this region was used as a representative value for constructing the calibration curves shown in the next paragraph.

**Figure 13:**
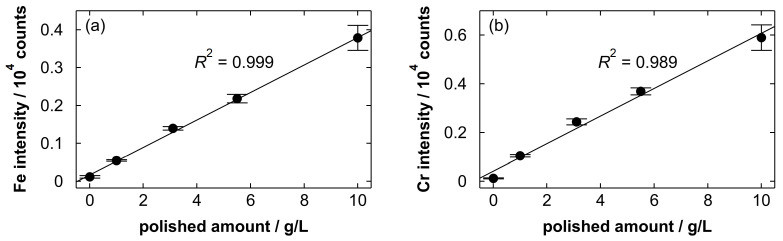
(**a**) The Fe intensity at 371.99 nm and (**b**) the Cr intensity at 359.35 nm plotted as a function of polished amount in the analysis of stainless steel electropolishing solutions through gas blowing. Each point represents the average intensity of three replicate measurements, and error bars indicate the standard deviation. A linear function fitted to the average intensity is shown together with its *R*^2^ value.

We analyzed stainless steel electropolishing solutions with different polished amounts of 0, 1.0, 3.1, 5.5 and 10.0 g/L through gas blowing. Figure 13 shows the Fe intensity at 371.99 nm and the Cr intensity at 359.35 nm plotted as a function of polished amount. Both the Fe and Cr intensity increased linearly with increasing polished amount, and the *R*^2^ values of the fitted linear functions were 0.999 and 0.989, respectively. The result suggests that the polished amounts of stainless steel products can be predicted by measuring the spectral line intensity of Fe or Cr. For the Fe line intensity, the LOD and LOQ were 1.7 and 5.6 g/L, respectively. The wavelength range to evaluate the background noise of the blank spectrum was from 371.85 to 372.24 nm (11 points). For the Cr line intensity, the LOD and LOQ were 1.1 and 3.7 g/L, respectively, which were obtained with the background noise range from 359.10 to 359.48 nm (11 points). Although the pulse energy was increased to obtain stronger signals, these emission lines were still relatively weak under the present conditions, resulting in high LOD and LOQ values. At the polished amount of 10.0 g/L, the RSD values of the Fe intensity and the Cr intensity evaluated based on three replicate measurements were 8.7% and 8.9%, respectively. At 10.0 g/L, the detectable changes, estimated using the same approach as for Nb, were approximately 1.4 and 1.5 g/L, respectively.

**Figure 14:**
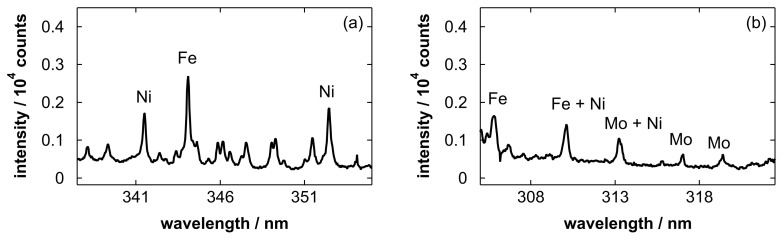
Emission spectra obtained after blowing stainless steel electropolishing solutions with a polished amount of 5.5 g/L in the wavelength ranges for the detection of (**a**) Ni and (**b**) Mo. Major emission lines of (**a**) Fe I at 344.06 nm, Ni I at 341.48 and 352.45 nm, (**b**) Fe I at 305.91, 309.99, 310.00, 310.03, and 310.07 nm (the last four lines overlap and are labeled together as Fe), Ni I at 310.16 and 313.41 nm, and Mo I at 313.26, 317.03, and 319.40 nm are labeled in the spectra.

We measured spectra in additional wavelength ranges to detect Ni and Mo. Figure 14 shows the resultant spectra. Although the signal intensity was relatively low, Ni and Mo emission lines were successfully observed. This suggests that the proposed method has potential for monitoring the dissolution behavior of each metal component during electropolishing, while the present study focused on the polished amount for determining the appropriate replacement timing of the solution. Optimizing the detection timing for each emission line can further improve sensitivity. For future work toward broader applicability and quantitative analysis of actual solutions, it will be necessary to investigate the effects of solution composition variations on the pretreatment and to evaluate calibration curves using metal concentrations independently determined by other analytical methods.

## 4. Conclusions

In this study, we introduced gas blowing as a non-contact sample pretreatment method for LIBS of microdroplet samples. After blowing Nb electropolishing solutions placed on porous Si, Nb emission lines were successfully detected under a low-energy condition, and the calibration curve for determining the polished amount exhibited good linearity. This suggests that gas blowing effectively suppresses excessive deposition of dry residue from highly concentrated electropolishing solutions while retaining an appropriate amount of sample for LIBS detection. Comparison with the wiping method highlighted an advantage of the non-contact method in which no impurities are introduced during pretreatment processes. We further extended this method to stainless steel electropolishing solutions, and Fe and Cr emission lines were successfully observed with linear correlations to the polished amount. The present results provide a basis for developing a LIBS-based analytical method for highly corrosive electropolishing solutions. The proposed method is expected to be applicable not only to electropolishing but also to various liquid-based industrial processes.

## Data Availability

The original contributions presented in this study are included in the article/Supplementary Materials. Further inquiries can be directed to the corresponding author.

## Supplementary Materials

The following supporting information can be downloaded at: https://www.mdpi.com/article/10.3390/ma19030637/s1. The spectral data used to generate the figures in the present study are provided as an Excel file.
